# 

**Published:** 1895-02-23

**Authors:** 


**Notices of Births, Deaths, and Marriages are inserted in this column at a charge of 2s. 6d. for an announcement not exceeding four lines.**

**Notice to Advertisers and Subscribers.**

All Advertisements, Orders for Copies of The Hospital and Business Communications should be addressed to **The Manager, NOT TO THE EDITOR,** The Hospital, 428, Strand, London, W.C.

The Cost of Subscription, post free, is as follows:—Per week, 2½d.; per quarter, 2s. 8d.; per half-year, 5s. 3d.; per annum, 10s. 6d. Foreign:—Per Annum, 15s. 2d.; payable, in all cases, in advance.

### NOTICE TO CORRESPONDENTS.

All MS., Letters, Books for Review, and other matters intended for the Editor should be addressed The Editor, The Lodge, Porchester Square, London, W.

The Editor cannot undertake to return rejected MS., even when accompanied by stamped directed envelope.

**"Burdett's Hospital Annual," 1895,**

Ready Early in March.

Sixth year of publication. About 1000 pp. Scarlet cloth, gilt lettered. Price 5s. A most complete guide to British, Colonial, Indian, and American Hospitals, Dispensaries, Nursing and Convalescent Institutions, and Asylums. A few copies of the "Annual" for 1894 may still be obtained on application to the Publishers, The Scientific Press (Limited), 428, Strand, London, W.C.

**NOTICE.—The Index for Vol. XVI. (ending September 24th, 1894) is on sale at the Office of this Journal, Price 2½d., post free.**

### SCRAPS AND GLEANINGS.

A NEW women's hospital has just been opened at Portland, in America.

A NEW wing at the Weymouth Sanatorium has been opened by Lady Ilchester.

THE Urban Ambulance Service of Paris has been officially handed over to the city authorities.

THE third congress of the French Obstetrical Society will be held on April 18th in Paris.

DURING the year more than 200 cases of accident have been treated at the Weymouth Royal Hospital.

THE trustees of the Newport and Monmouthshire Infirmary have been credited with £500 from the executors of the late Mr. Whitehouse.

AT a late meeting of the Charity Organisation Society there was a strong and general protest urged against employing children to beg in the streets on behalf of charitable societies.

THE annual meeting of the Jenny Lind Hospital, Norwich, took place recently. An increase in donations and bequests is shown in the report, but there is a falling off in the subscription list.

WHEREAS, at the close of 1893 there was a deficit of £102 on the accounts of the Blackheath and Charlton Cottage Hospital, the committee have commenced 1895 with a surplus of £95.

THE special effort made during the year by the ladies and the General Committee of the Wirral (Liverpool) Children's Hospital in behalf of the finances of the institution realised no less a sum than £500.

MADAME SARRANTE, in advocating bicycling for women, has certainly endangered her cause with a large number of women by stating that riders after a short time find it necessary to enlarge their corsage.

A LIFE pension of £25 per annum has been awarded to Mrs. Page, the matron of the Hunstanton Convalescent Home. This proposal was made in recognition of the matron's valuable services to the institution.

BESIDES certain changes in the medical arrangements of the Cardiff Infirmary, this name of the institution—the Cardiff Infirmary—has been definitely substituted for the Glamorganshire and Monmouthshire Infirmary.

A PLOT of land has been presented to the Halstead Cottage Hospital through the generosity of Mr. Coutauld. This gift will prove a great boon to the patients, as the land adjoins the hospital. It will also materially increase the dimensions of the garden.

STATISTICS on the relation between oyster eating and typhoid are still coming in, and the areas from which the information are collected continue to spread. Italy is now proving a fruitful source from which examples confirming the theory can be drawn.

MEASURES are frequently being taken to allay the increasing prevalence of drunkenness in St. Petersburgh. The governor of that city has now ordered the names and addresses of the persons found drunk in the public streets to be posted in fixed public places, and duly entered in the official gazette.

THE Ladies' Samaritan Society connected with the National Hospital for the Paralysed and Epileptic, Bloomsbury, has issued an appeal, in which it calls attention to the urgent need of funds for that society. It will be necessary, unless help is forthcoming, to further curtail the relief given to necessitous patients.

THE maintenance of the Brentwood District Cottage Hospital has been carried on in this, the first year of its existence, through the support of eight of the leading inhabitants of the neighbourhood who offered to guarantee £25 each. The committee now start on the new year's work with a satisfactory balance in hand.

SIR RICHARD PAGET, in reviewing the work of the Shepton Mallet District Hospital, referred to the excellent work the institution was doing. He also made reference to a handsome donation guaranteed to him for the working expenses of the hospital in the instance of Mr. Richard Burt, who has contributed £100.

THE statement of receipts and expenditure at the annual meeting of subscribers to the Princess Alice Memorial Hospital, Eastbourne, shows the latter to be very heavy in comparison with the former, the adverse balance having reached the sum of £600. This deficiency it is proposed to meet by opening a "special" fund at the bank.

THE urgent needs of the Brighton Hospital for Women and Children were dwelt upon at the sixty-fourth annual meeting. The chairman of the committee remarked that if the authorities were unable to balance their income and expenditure there was a danger that they might be compelled, as they were once before, to close their wards!

THROUGH the generosity of Miss L. da Costa the committee of the Sussex Eye Hospital, at the sixty-second annual meeting recently held, were enabled to present a balance-sheet of a far more satisfactory character than has been the case of recent years. To clear off a deficit Miss da Costa early in the year gave the hospital £200, making up, with her previous donations, the munificent sum of £900.

THERE has been a marked increase in the appreciation of the Glasgow Eye Infirmary lately, 102 students' names having been on the books during the past year. Very important pathological work has been done, and it is reported that the largest source of injury to the eye involving loss of sight is due to accidents in public works, the second largest being in connection with the opening of bottles of aerated waters.

### BOOKS RECEIVED.

P. BLAKISTON AND SON, PHILADELPHIA.

"Surgical Nursing." By Bertha M. Voswinkel.

TERRIS AND CO., BRISTOL.

"Pocket Therapeutic Notes." Second Edition. 1894.

WELLS GARDNER, DARTON, AND CO.

"How to Nurse in Our Homes." By A. M. Alexander.

T. FISHER UNWIN.

"Good Reading about Many Books." Mostly by their Authors.

**Periodicals and Pamphlets.**—Cornhill Magazine, New Review, Cassell's Family Magazine, The Tri-State Medical Journal, Indian Medico-Chirurgical Review, Dissemination of Infectious Diseases by Boarding Schools, Modern Methods of Investigating Female Pelvic Disease, Edinburgh Medical Missionary Society, The Humanitarian, The Practitioner, The Asclepiad, Die Kritik, Dublin Journal of Medical Science, Charlotte Medical Journal, Clinical Journal, Public Health, Strand Musical Magazine, Strand Magazine, Picture Magazine, Strand Novelettes, Round the World, Medical Pioneer, Christian World, Children's League of Pity, Guy's Hospital Gazette, The Bookseller, Proceedings of Society for Study of Inebriety, Medical Chronicle, American Medico, Surgical Bulletin, American Bookmaker, Industries and Iron.

### THE STUDENT OF MEDICINE.

### APPOINTMENTS.

H. W. Arbuckle, M.D.Aberd., reappointed Medical Officer to the Thorne District Council. G. T. C. Barber, M.R.C.S., L.R.C.P.Lond., appointed Medical Officer to the Parish of Birmingham Workhouses. Dr. Barnes, appointed Medical Officer of Health to the Chard Town Council. L. Bostock, L.R.C.P., L.R.C.S.Edin., appointed Medical Officer for the 4th District of the Liskeard Union. R. J. Boustead, L.R.C.P. L.R.C.S.Edin., reappointed Medical Officer of Health to the Haltwhistle Rural District Council. Dr. H. Cameron, appointed Medical Officer to the Parochial Board of Rosskeen. J. B. Coumbe, F.R.C.S.Edin., M.D.Brux., L.R.C.P.Edin., appointed Medical Officer for the No. 9 District of the Bedminster Union. A. K. Crossfield, L.R.C.P., L.R.C.S.Edin., reappointed Medical Officer for the Dittisham District of the Totnes Union. J. Elias, M.R.C.S.Eng., D.P.H.Camb., appointed Medical Officer of Health to the Neath Town Council. G. A. Ferraby, L.R.C.P.Lond., M.R.C.S.Eng., appointed Medical Officer for the Fourth and Fifth Districts of the Parish of Birmingham. E. S. Greensill, L.R.C.P.Edin., M.R.C.S.Eng., appointed Medical Officer for the Martley District of the Martley Union. L. G. D. Jones, M.R.C.S., L.R.C.P.Lond., appointed Medical Officer for the First and Second Districts of the Parish of Birmingham. J. Johnston, M.D., M.Ch.Edin., L.S.A.Lond., Honorary Surgeon to Bolton Infirmary, appointed Public Vaccinator for the Great Bolton District. T. Lakeman, L.R.C.P. Lond., M.R.C.S.Eng., reappointed Medical Officer for the Ugborough District of the Totnes Union. L. H. Pegler, M.D., M.R.C.S., appointed Assistant Registrar to the Central London Throat and Ear Hospital. J. R. I. Raywood, L.R.C.P.Lond., M.R.C.S.Eng., L.S.A.Lond., appointed Honorary Surgeon to the Montgomeryshire Infirmary, Newtown, N. Wales. K. R. Smith, M.D.Lond., reappointed Medical Officer for the Halwell District of the Totnes Union. A. Stanley, M.B.Lond., appointed Assistant Physician to the Manchester and Salford Hospital for Skin Diseases. J. Tennant, M.A., M.B., C.M.Edin., appointed House Surgeon to the Victoria Hospital, Folkestone. H. Ubsdell, M.R.C.S.Eng., L.S.A., reappointed Medical Officer for the Staverton and Rattery District of the Totnes Union. R. B. Wild, M.D.Lond., M.Sc.Vict., appointed Honorary Physician to the Manchester and Salford Hospital for Skin Diseases. J. F. Wright, L.R.C.P.Lond., M.R.C.S.Eng., appointed Medical Officer to the Western Division of Great Bolton. E. S. Yonge, M.B., C.M.Edin., appointed Honorary Surgeon to the Chorlton-on-Medlock Dispensary, Manchester. A. Young, L.R.C.P.Lond., M.R.C.S.Eng., appointed Certifying Factory Surgeon for Hampstead. Mrs. Caroline Keith, L.R.C.S., L.R.C.P.Ed., ap-

### THE STUDENT OF MEDICINE—*continued.*

pointed Anæsthetist to the Chelsea Hospital for Women. Mr. A. J. Stuck, M.R.C.S., L.R.C.P., appointed Resident Medical Officer to the Chelsea Hospital for Women.

#### VACANCIES.

The following vacancies are announced this week, the amount of salary in each case being given after the name of the institution:

**Resident Medical Officers.**

Guest Hospital, Dudley.—Resident Assistant House Surgeon. Appointment for six months. Board, lodging, and washing provided in the hospital; no salary. Applications to the Secretary by February 27th.

Paddington Green Children's Hospital, W.—House Surgeon. Salary £50, with board and residence. Appointment from April 1st to November 7th. Also Surgeon to the Out-patients. Applications to the Secretary by March 1st.

Royal Albert Edward Infirmary and Dispensary, Wigan.—Junior House Surgeon; doubly qualified. Salary £80 per annum, with apartments and rations. Applications to Will Taberner, General Superintendent and Secretary, by February 27th.

St. Peter's Hospital, Henrietta Street, Covent Garden, W.C.—House Surgeon. Appointment for six months. Salary at the rate of 50 guineas a year, with board, lod ing, and washing, and an allowance for wine, &c. Applications to the Secretary by March 1st.

Worcester County and City Lunatic Asylum.—Third Assistant Medical Officer; unmarried. Salary commences at £100 per annum, with board, lodging, and washing. Applications to Dr. Cooke, The Asylum, Powick, near Worcester, by March 1st.

**Non-Resident Medical Officers.**

St. George's Hospital, S.W.—Surgeon and Assistant Surgeon; must be F.R.C.S.Eng. Applications to the Secretary by March 1st.

Westminster Hospital, Broad Sanctuary, S.W.—Fourth Assistant Physician, must be Fellow or Member of the Royal College of Physicians of London, not practising pharmacy and midwifery. Applications to the Secretary before February 26th.

#### EDINBURGH TRIPLE QUALIFICATION.

FINAL EXAMINATION.

MEDICINE.

(Three questions only to be answered.)

1. What are the two varieties of angina pectoris? Mention the forms of heart disease in which angina pectoris is most common, and give a full description of a paroxysm.
2. What are the causes, symptoms, physical signs, and consequences of spasmodic asthma. Describe an attack.
3. Give the causes, symptoms, and course of pseudo-hypertrophic paralysis.
4. Give the causes, symptoms, and course of diphtheria. In what various ways may it prove fatal?

THERAPEUTICS.

(All the questions to be answered, including the prescription.)

1. Describe how you would treat a patient threatened with cardiac asystole, as shown by extreme cardiac dyspnoea, feeble and irregular pulse, &c.
2. Give the appropriate treatment—(1) for the night sweats; and (2) for the diarrhœa of advanced phthisis.
3. How would you treat a case of acute catarrh of the stomach produced by alcoholism?

PRESCRIPTION.

(To be written in unabbreviated Latin.)

Prescribe a mixture containing digitalis and squill for a case of mitral disease of the heart with bronchitis.

